# Construction of a robust sepsis prognostic classifier based on E3 ubiquitin ligase-related genes

**DOI:** 10.3389/fmolb.2026.1726356

**Published:** 2026-02-17

**Authors:** Haiyan Xue, Lihe Chen, Xiujuan Zhao, Fengxue Zhu

**Affiliations:** 1 Department of Critical Care Medicine, Peking University People’s Hospital, Beijing, China; 2 Division of Medicine, University College London, London, United Kingdom

**Keywords:** immune cells, machine learning, predict model, sepsis, ubiquitination

## Abstract

Sepsis is a life-threatening disease with high mortality and one of the leading causes of death worldwide. Although studies have shown that ubiquitylation is closely related to the occurrence and development of sepsis, the prognostic and diagnostic value of ubiquitylation-related genes in sepsis remains unclear. In this study, we obtained sepsis datasets from the Gene Expression Omnibus (GEO) database and identified ubiquitylation-related genes from the Ubiquitin and Ubiquitin-like Conjugation Database (iUUCD). We identified 159 differentially expressed genes related to ubiquitylation between sepsis patients and healthy individuals, and the prognosis of sepsis subgroups distinguished by these ubiquitylation genes showed significant differences, demonstrating the importance of the ubiquitylation phenotype in sepsis. To explore the prognostic value of ubiquitination-related genes in sepsis, we constructed a ubiquitylation-related score (URS) through LASSO analysis, random forest, and Cox regression analysis. Importantly, we validated the reliability of this model in both the GEO database cohort and the external cohort data from our unit. Finally, we identified LTB and TCP11L2 as key ubiquitylation-related genes, explored their expression patterns and potential biological context through single-cell RNA sequence analysis, and validated their expression and diagnostic value using patient blood samples. Our study suggests a link between ubiquitylation and sepsis, which can be used as a potential biomarker to guide the diagnosis, treatment, and prognosis of sepsis and proposes new ideas for future clinical research.

## Introduction

Sepsis is a life-threatening syndrome of organ dysfunction caused by a dysregulated host response to infection (Singer et al., 2016), characterized by complex and multifactorial pathophysiology (Lelubre and Vincent, 2018; Meyer and Prescott, 2024). Globally, it accounts for approximately 20% of all annual deaths and remains one of the leading causes of mortality in intensive care units (Vandewalle and Libert, 2022; Rudd et al., 2020; Li et al., 2018). One important reason for this persistently high mortality is the lack of reliable biomarkers for early diagnosis and risk stratification (Wang et al., 2020). Although numerous predictive models have been developed using clinical parameters (Sahu et al., 2022) or molecular features (Chen et al., 2024), their clinical utility remains limited (Yu et al., 2023). Thus, identifying novel biomarkers and developing effective prognostic tools is a major priority in sepsis research (Kalil and Sweeney, 2018; Pierrakos and Vincent, 2010; Reinhart et al., 2012) and forms the central aim of this study.

Ubiquitin (Ub) is a highly conserved 76–amino acid protein present in all eukaryotic cells (Goldstein et al., 1975). Ubiquitination, a post-translational modification mediated by E1 activating enzymes, E2 conjugating enzymes, and E3 ligases (Popovic et al., 2014; Allan and Phillips, 2017), regulates not only protein degradation but also diverse cellular functions (Hershko and Ciechanover, 1998). Dysregulated ubiquitination has been implicated in multiple pathological conditions (Cockram et al., 2021). In sepsis, it participates in key processes such as autophagy (Chen et al., 2019; Li and Wang, 2023), oxidative stress, inflammasome activation, and immune regulation (Wang et al., 2024; Wang et al., 2023). Given its central role, the ubiquitin system has emerged as a potential therapeutic target in sepsis (Shao et al., 2023; Dao et al., 2024; Liu et al., 2021). Nevertheless, due to its complexity and heterogeneity, the precise mechanisms and clinical implications of ubiquitination in sepsis remain incompletely understood (Yau and Rape, 2016).

Based on the established role of ubiquitination in sepsis-related immune dysregulation, and given that E3 ligases represent the largest and most central component of the ubiquitination system, dictating target specificity, we hypothesized that E3 ubiquitin ligase-related genes could serve as robust prognostic biomarkers for sepsis. To test this hypothesis, we systematically analyzed sepsis transcriptomic datasets and integrated E3 ubiquitin ligase–related genes to identify prognostically relevant molecular signatures. By applying multiple machine learning algorithms, we developed a robust ubiquitination-related score (URS) and validated its predictive performance across public cohorts and an independent clinical cohort. Furthermore, we explored the biological roles of key candidate genes through single-cell RNA sequencing and confirmed their diagnostic value in patient samples using RT-qPCR. Our findings support this hypothesis and may provide new insights into the potential contribution of ubiquitination to sepsis pathogenesis, and highlight potential biomarkers and prognostic tools that may improve patient stratification and guide clinical decision-making.

## Materials and methods

### Download and process data

We included 997 E3 ubiquitination-related genes from the iUUCD database (Zhou et al., 2018) (http://iuucd.biocuckoo.org/) for research. We downloaded sepsis whole blood sample transcriptome sequencing-related datasets from the Gene Expression Omnibus (GEO, https://www.ncbi.nlm.nih.gov/geo/). The training cohort GSE65682 comprised 42 healthy controls and 760 sepsis subjects with complete survival status data. We validated the prognostic value of the prognostic model using the GSE95233 dataset, which also contained follow-up information. Additionally, peripheral blood samples from 10 healthy individuals and 30 sepsis patients from our unit were collected for RNA sequencing for external validation of the model. Moreover, seven other sepsis-related datasets without prognostic information (GSE54514, GSE131761, GSE137340, GSE236713, GSE28750, GSE57065, GSE69528) were selected to confirm the diagnostic value of the model. To further investigate the potential mechanisms of key molecules in immune cells, we downloaded GSE175453 from GEO, which contained scRNA-seq data from whole blood samples collected from five healthy controls and four sepsis donors.

### Study population

We prospectively collected peripheral blood samples from 30 sepsis patients and 10 healthy controls for transcriptome sequencing to serve as an external validation for the model. Additionally, we prospectively collected samples from 20 sepsis patients and 10 healthy controls for real-time quantitative Polymerase Chain Reaction (RT-qPCR) experiments to validate the expression of key genes and their predictive value. All patients were recruited from the intensive care units of Peking University People’s Hospital from January 2023 to December 2024. The inclusion criteria were based on the Sepsis-3.0 definitions, which require a suspected infection plus a Sequential Organ Failure Assessment (SOFA) score of 2 or higher. Healthy controls were selected from individuals undergoing routine health check-ups who did not have any signs or symptoms of infection or inflammation.

### Transcriptome sequencing

Peripheral whole blood samples were collected from all participants at the time of enrollment. Total RNA was extracted using the TRIzol® Reagent following the manufacturer’s instructions. RNA samples were detected based on the A260/A280 absorbance ratio with a Nanodrop ND-2000 system (Thermo Scientific, United States), and the RIN of RNA was determined by an Agilent Bioanalyzer 4,150 system (Agilent Technologies, CA, United States). Only qualified samples will be used for library construction. Paired-end libraries were prepared using a ABclonal mRNA-seq Lib Prep Kit (ABclonal, China) following the manufacturer’s instructions. Finally, sequencing was performed with an Illumina MGISEQ-T7 instrument. The transcriptomic data have been uploaded to the GEO database under accession number GSE294985.

## Identification of ubiquitination-related differentially expressed genes (DEGs)

We used the “limma” package to identify genes with differential expression (P value <0.05 and |LogFC| >1) between normal and sepsis patient blood samples, and extracted the DEGs as candidate genes. Further, we screened out the ubiquitination-related genes from the candidate genes as ubiquitination-related DEGs for subsequent analysis. Subsequently, these ubiquitination-related DEGs were subjected to functional enrichment analysis based on the Metascape database (www.metascape.org/), with P < 0.05 considered as significantly enriched.

### Patient subtyping based on ubiquitination-related DEGs

Consensus clustering was performed using the “ConsensusClusterPlus” package in R based on the prognostic ubiquitination-related DEGs. The optimal number of clusters was determined by analyzing the cumulative distribution function (CDF) curves. Subsequently, principal component analysis (PCA) was conducted using the “prcomp” function in R to confirm the reliability of the clustering. Kaplan-Meier survival analysis and log-rank tests were performed using the survival package in R to analyze survival differences among different groups and to display the survival status of all patient samples within 25 days. These results were further validated in the GSE95233 dataset. Next, we identified prognostic DEGs between different sepsis subtypes as Sub-DEGs. Functional enrichment analysis of these genes was conducted using the Reactome pathway database through the clusterProfiler 4.8.2 package in R. Enrichment with P < 0.05 was considered significant.

### Establishment of ubiquitination-related sepsis prognostic model

Among the Sub-DEGs, we employed three distinct machine learning algorithms to screen for robust prognostic genes associated with mortality in sepsis patients. Detailed parameters and cross-validation strategies for each algorithm were as follows:

1. LASSO (Least Absolute Shrinkage and Selection Operator) Regression: We applied LASSO regression using the glmnet package (version 4.1–8) in R. A 10-fold cross-validation was performed to determine the optimal regularization parameter (λ). The λ value that yielded the minimum partial likelihood deviance (using the lambda. min criterion) was selected. This process automatically performed feature selection by shrinking the coefficients of non-informative genes to zero.
2. CoxBoost (Cox regression with likelihood-based boosting): The CoxBoost package (version 1.4) was utilized. To prevent overfitting and determine the optimal number of boosting steps, we performed an internal 10-fold cross-validation (type = “verweij”, stepno = 100). The optimal step number was chosen as the one that maximized the cross-validated partial log-likelihood (CVPL). The penalty parameter penalty was set to the default value (9 * sum (status)), where status is the event indicator.
3. Random Survival Forest (RSF): We used the randomForestSRC package (version 3.2.3) to build a RSF model. The key parameters were set as follows: number of trees (ntree) = 1,000; number of variables randomly sampled as candidates at each split (mtry) was set to the default (square root of the number of predictor variables). Variable importance (VIMP) for mortality prediction was calculated using permutation error. Internal validation was performed using out-of-bag (OOB) error estimation, which provides an unbiased estimate of the model’s prediction error.

Based on these three machine learning algorithms, we selected Sub-DEGs that were identified by at least two of the algorithms for following analysis. Further, an ubiquitination-related risk score (URS) was established using multivariable Cox regression on these consensus genes. Patients were divided into high- and low-risk groups according to the median value of the URS, and differences in prognosis were analyzed and validated in the GSE95233 dataset and the external cohort GSE294985.

### Meta-analysis

To enhance the clarity of the diagnostic and prognostic value of the URS, we utilized the “meta” package in R to combine the odds ratios (OR) and hazard ratios (HR) obtained from multiple studies. Based on the degree of heterogeneity determined by the I^2^, a fixed-effects or random-effects model was used to extract and summarize the data from each study.

### Assessment of URS diagnostic efficacy

We used the pheatmap package to illustrate the distribution of other clinical features under URS stratification in GSE65682, GSE95233 and GSE294985. Additionally, univariable and multivariable Cox regression analyses were employed to identify independent prognostic factors and to evaluate the independent prognostic performance of the URS. Moreover, the diagnostic capability of the URS and its model genes for sepsis was assessed by constructing receiver operating characteristic (ROC) curves using the “pROC” package in eight datasets to demonstrate the diagnostic ability of the URS for the occurrence of sepsis. Subsequently, we integrated the URS with other clinical information to establish a clinical Nomogram model. The calibration plot was used to assess the fit of the model scores, and finally, ROC curves were employed to display the predictive performance of the URS and the Nomogram score for patient prognosis.

### Single-cell sequencing data analysis

We used the “Seurat” R package to preprocess and analyze the single-cell RNA sequencing (scRNA-seq) data of sepsis patients. The “NormalizeData” function of the “Seurat” package was used to normalize the scRNA-seq data, with the normalization method set to “LogNormalize.” The normalized data were then converted into a Seurat object. The percentage of mitochondrial or ribosomal genes was calculated, and low-quality cells were excluded to ensure quality control (QC). We excluded samples with fewer than 200 or more than 3,000 genes, as well as those with a ribosomal RNA proportion exceeding 20%. Subsequently, using the FindVariableFeatures function of the Seurat package, the top 3,000 genes were selected as the most important variable features to standardize the scRNA-seq data for each cell. Additionally, we executed the ScaleData and RunPCA functions to obtain the number of principal components (PCs) based on the Seurat object. We used “UMAP (Uniform Manifold Approximation and Projection)” dimensionality reduction to further summarize the principal components. Finally, automatic annotation was performed using the “SingleR” package, and the Idents and DimPlot functions were utilized to visualize the major cell types or subtypes. Subsequently, we explored the expression differences and functional enrichment of key genes in the URS model between normal and sepsis samples. Next, we identified URS high-risk and low-risk cell subclusters, demonstrated the overall URS score differences among different cells, and analyzed the functional enrichment differences between URS high-risk and low-risk cell subclusters.

### Real-time quantitative PCR (RT-qPCR)

Total RNA was isolated using the TRIzol™ Plus RNA Purification Kit, followed by reverse transcription with a dedicated cDNA synthesis kit. Quantitative PCR was carried out on the AriaMx Real-Time PCR System utilizing SYBR® Green-based detection. Relative gene expression levels were calculated using the 2^ΔΔCt method, with GAPDH serving as the internal control. Results were normalized to the control group (set as 1). Primer sequences used in this analysis are provided in Supplementary Table S1.

### Statistical analysis

All data analyses were performed using R software (version 4.3.1). Differences between two groups were assessed using the Wilcoxon rank-sum test, while comparisons among multiple groups were carried out using the Kruskal-Wallis test. Correlation analysis was conducted based on Spearman’s rank correlation method. A p-value below 0.05 was deemed statistically significant. Significance thresholds were represented as follows: *p < 0.05; **p < 0.01; ***p < 0.001.

## Results

### Identification of ubiquitination-related DEGs associated with sepsis

**FIGURE 1:**
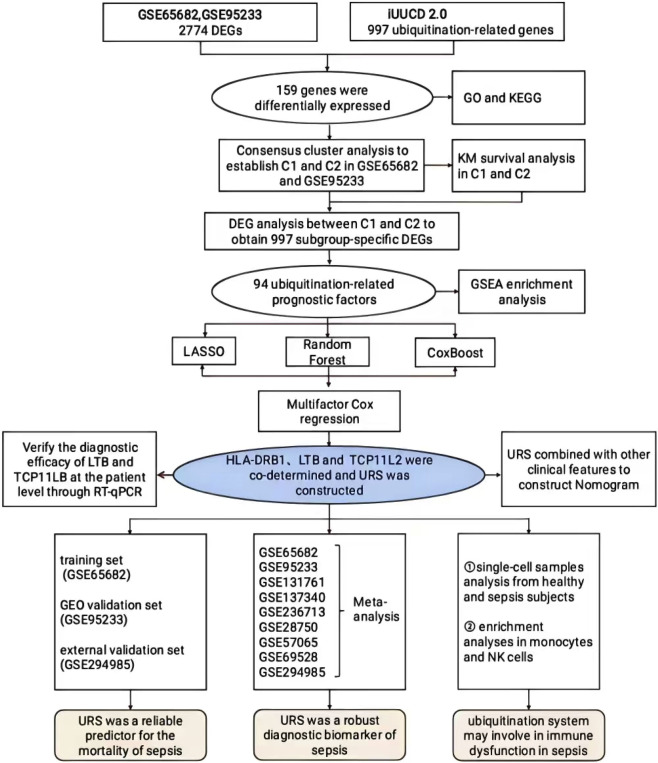
The workflow of this study.

**FIGURE 2:**
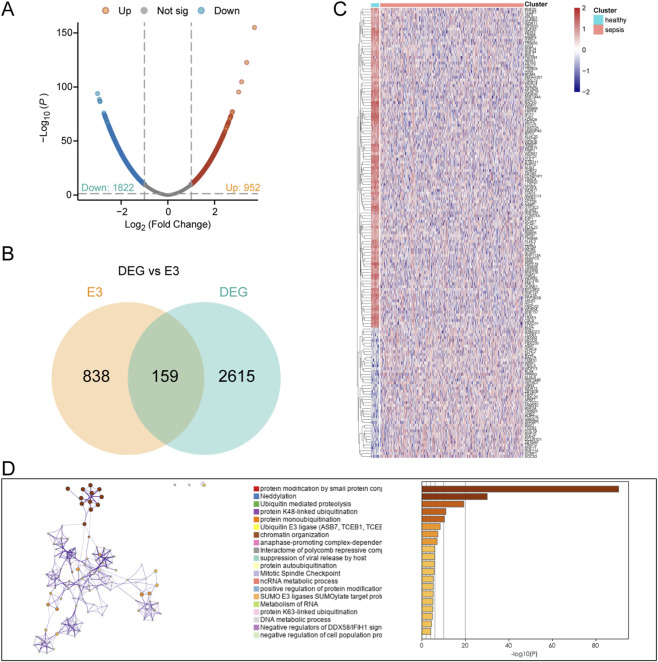
Identification of ubiquitination-related DEGs associated with sepsis. **(A)** Volcano plot of DEGs between sepsis and healthy individuals. **(B)** Venn plot of the DEGs and E3 ubiquitination-related genes. **(C)** Heatmap of ubiquitination-related DEGs between sepsis and healthy individuals. **(D)** Functional enrichment of ubiquitination-related DEGs.

The graph abstract of our study is shown in Figure 1. First, we chosen GSE65682 as the training set and identified a total of 2,774 differentially expressed genes (DEGs) between sepsis and healthy samples, including 952 up-regulated genes and 1822 down-regulated genes (Figure 2A). After intersecting these DEGs with the 997 E3 ubiquitination-related genes collected from the iUUCD database, we obtained 159 ubiquitination-related DEGs (Figure 2B). Among these 159 ubiquitination-related DEGs, 46 were up-regulated and 113 were down-regulated in sepsis patients (Figure 2C). Subsequently, we performed functional enrichment analysis of these ubiquitination-related DEGs using the Metascape database. The results showed that these genes were primarily associated with multiple ubiquitination processes, such as protein modification, neddylation, and proteolysis (Figure 2D).

### Identification of patient subtypes based on the ubiquitination-related DEGs

**FIGURE 3:**
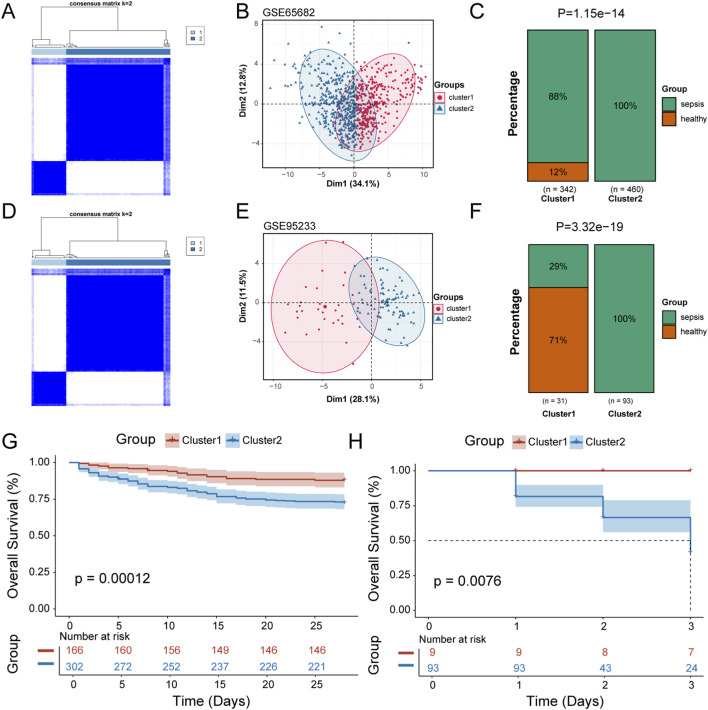
Identification of patient subtypes based on the ubiquitination-related DEGs **(A)** GSE65682 was divided into two subgroups by unsupervised clustering. **(B)** PCA analysis of the two subgroups in GSE65682. **(C)** Distribution of disease status in the two subgroups of GSE65682. **(D)** GSE95233 was divided into two subgroups by unsupervised clustering. **(E)** PCA analysis of the two subgroups in GSE95233. **(F)** Distribution of disease status in the two subgroups of GSE95233. **(G)** KM survival curves between the two subgroups in GSE65682. **(H)** KM survival curves between the two subgroups in GSE95233.

We first performed univariable regression analysis on the ubiquitination-related DEGs, identifying 37 genes that significantly impacted patient prognosis (Supplementary Table S2). Consensus clustering analysis was then conducted on these genes, dividing the 802 samples in the GSE65682 cohort into two subgroups (Cluster 1 and Cluster 2) (Figure 3A). Principal Component Analysis (PCA) demonstrated good separation between these two subgroups (Figure 3B). Analysis of the characteristics of patients in these two groups revealed that sepsis patients were predominantly concentrated in Cluster 2 (p = 1.15e-14) (Figure 3C). Consensus clustering was also performed in the validation cohort GSE95233, where patients were similarly divided into two subgroups (Cluster 1 and Cluster 2) (Figure 3D). PCA showed good separation (Figure 3E), with sepsis patients mainly concentrated in Cluster 2 (p = 3.32e-19) (Figure 3F). Kaplan-Meier (KM) survival analysis was conducted for patients in the two subgroups, revealing significant prognostic differences between the subgroups in both GSE65682 (Figure 3G) and GSE95233 (Figure 3H), with p-values of 0.00012 and 0.0076, respectively. These findings suggest a clear correlation between the ubiquitination status of patients and sepsis prognosis.

### Establishment of ubiquitination-related sepsis prognostic model

**FIGURE 4:**
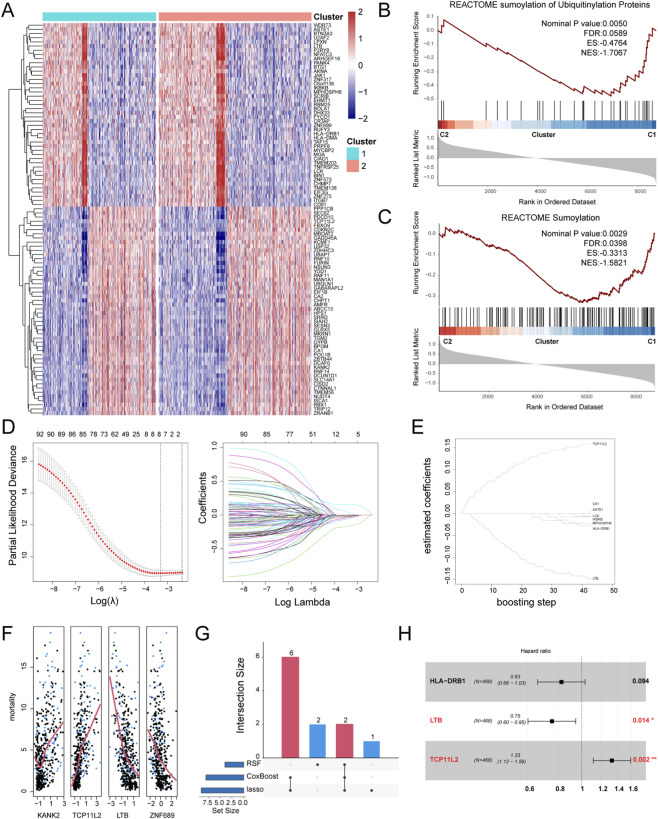
Establishment of Ubiquitination-Related Sepsis Prognostic Model. **(A)** Heatmap of the prognostic sub-DEGs between two subgroups. **(B)** GSEA enrichment analysis of Sumoylation of Ubiquitinylation Proteins pathway. **(C)** GSEA enrichment analysis of Sumoylation pathway. **(D)** Screening prognostic genes using the LASSO algorithm. **(E)** Screening prognostic genes using the CoxBoost algorithm. **(F)** Screening prognostic genes using the RSF algorithm. **(G)** Upset plot of the machine learning results. **(H)** Forest plot of the multivariable Cox regression analysis.

We further conducted DEG analysis between the two subgroups, obtaining 997 subgroup-specific DEGs. After univariate regression analysis of these genes, 94 sub-DEGs with prognostic value were identified (Figure 4A; Supplementary Table S3). Reactome pathway enrichment analysis indicated that these 94 sub-DEGs were significantly enriched in ubiquitination-related pathways: Sumoylation of Ubiquitinylation Proteins (Figure 4B) and Sumoylation (Figure 4C). We then screened key prognostic genes from these 94 sub-DEGs using various machine learning methods. Using the LASSO algorithm, we identified 9 important ubiquitination-related prognostic factors: CA1, ZNF317, TCP11L2, LCK, IKBKB, ASTE1, HLA-DRB1, LTB, and MPHOSPH8 (Figure 4D). Using the CoxBoost algorithm, we identified 8 important ubiquitination-related prognostic factors: CA1, TCP11L2, LCK, IKBKB, ASTE1, HLA-DRB1, LTB, and MPHOSPH8 (Figure 4E). Using the Random Survival Forest (RSF) algorithm, we identified 4 important ubiquitination-related prognostic factors: KANK2, TCP11L2, LTB, and ZNF689 (Figure 4F). We selected sub-DEGs that were identified by at least two of the algorithms for further analysis, resulting in a total of 8 key sub-DEGs (Figure 4G). We performed multivariable Cox regression analysis on these 8 genes and constructed the following prognostic model for sepsis patients based on their respective Coefficients values: URS = HLA-DRB1 × (-0.19) + LTB × (-0.28) + TCP11L2 × (0.27). Among them, LTB and TCP11L2 demonstrated independent prognostic ability (Figure 4H).

### Validation of the reliability of the URS

**FIGURE 5:**
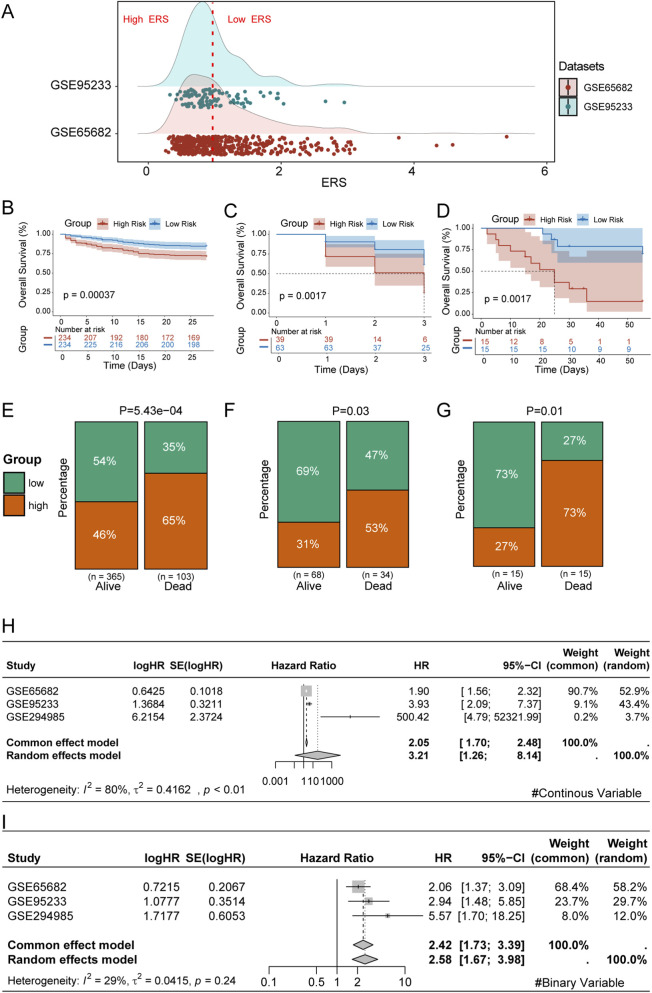
Validation of the Reliability of the URS. **(A)** Ridge plot of URS score distribution in the training and validation sets. **(B)** KM analysis of sepsis patients in the High and Low URS groups in GSE65682. **(C)** KM analysis of sepsis patients in the High and Low URS groups in GSE95233. **(D)** KM analysis of sepsis patients in the High and Low URS groups in GSE294985. **(E)** Bar chart of the distribution of sepsis patients in the High and Low URS groups in GSE65682. **(F)** Bar chart of the distribution of sepsis patients in the High and Low URS groups in GSE95233. **(G)** Bar chart of the distribution of sepsis patients in the High and Low URS groups in GSE294985. **(H)** Meta-analysis based on the URS score. **(I)** Meta-analysis based on the URS risk groups.

Subsequently, we validated the prognostic value of the URS in multiple datasets, including the training set (GSE65682), the GEO validation set (GSE95233), and the external validation set from our unit (GSE294985). Based on the median value of the URS in the training set GSE65682, all sepsis patients were divided into High URS risk and Low URS risk groups (Figure 5A). We then compared the survival of patients in the high and low URS risk groups across the three different datasets. The results showed significant survival differences between the high and low URS risk groups in all three datasets (Figures 5B–D). Consistently, percentage bar charts showed that in all three datasets, the proportion of High URS was higher among dead patients, while it was lower among surviving patients (Figures 5E–G). Subsequently, we conducted a meta-analysis based on the URS risk score and risk groups. The results showed that URS is an important prognostic predictor, with a pooled HR of 3.21 (95% CI = 1.26–8.14) for URS score (Figure 5H) and a pooled HR of 2.58 (95% CI = 1.67–3.98) for the URS risk groups (Figure 5I).

We also investigated the distribution of clinical characteristics among patients with different URS statuses. Based on the available clinical information, we generated clinical information heatmaps for GSE65682 (Supplementary Figure S1A), GSE95233 (Supplementary Figure S1B), and GSE294985 (Supplementary Figure S1C). The results indicated significant differences in pneumonia among different URS groups. This observation suggests that the URS may have varying associations with specific infection etiologies, hinting at the underlying heterogeneity of sepsis. In addition, we performed univariable and multivariable regression analyses incorporating clinical information to assess the independent prognostic ability of URS. The results showed that URS could serve as an independent prognostic factor in GSE65682 (Supplementary Figures S2A,B), GSE95233 (Supplementary Figures S2C,D), and GSE294985 (Supplementary Figures S2E,F).

Considering the impact of patients’ clinical characteristics on prognosis, in order to establish a more robust clinical prognostic model, we combined the URS with clinical information to construct a Nomogram model (Supplementary Figure S3A). The calibration curve indicated a good fit with the actual prognosis of patients (Supplementary Figure S3B). Therefore, we used ROC curves to evaluate the performance of the URS score (Supplementary Figure S3C) and the Nomogram score (Supplementary Figure S3D) in predicting patient prognosis. We found that compared with the prognostic ability of the URS score (AUC = 0.656), the predictive ability of the Nomogram score was significantly improved (AUC = 0.706).

### The diagnostic capability of the URS score for sepsis patients

**FIGURE 6:**
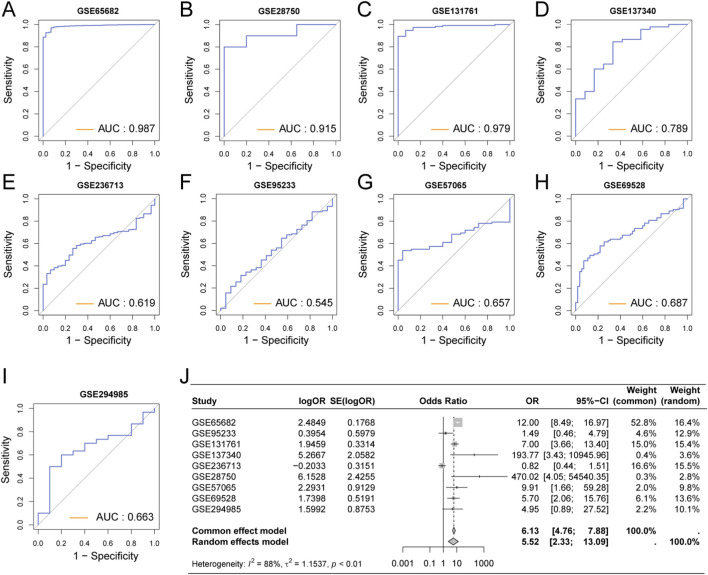
Assessment of the Diagnostic Efficacy of URS for Sepsis Patients. **(A)** The diagnostic ROC curve of URS for sepsis in GSE65682. **(B)** The diagnostic ROC curve of URS for sepsis in GSE28750. **(C)** The diagnostic ROC curve of URS for sepsis in GSE131761. **(D)** The diagnostic ROC curve of URS for sepsis in GSE137340. **(E)** The diagnostic ROC curve of URS for sepsis in GSE236713. **(F)** The diagnostic ROC curve of URS for sepsis in GSE95233. **(G)** The diagnostic ROC curve of URS for sepsis in GSE57065. **(H)** The diagnostic ROC curve of URS for sepsis in GSE69528. **(I)** The diagnostic ROC curve of URS for sepsis in GSE294985. **(J)** Meta-analysis of the diagnostic efficacy of URS for sepsis.

To evaluate the role of the URS in the diagnosis of sepsis, we included multiple datasets containing sepsis patients and healthy controls, and drew ROC curves to assess the diagnostic performance. We found that in GSE65682 (Figure 6A), GSE28750 (Figure 6B), and GSE131761 (Figure 6C), the AUC values were all greater than 0.9, demonstrating excellent diagnostic efficacy. In other datasets, the AUC values ranged from 0.5 to 0.8 (Figures 6D–I). We conducted a meta-analysis of the above datasets, which showed a pooled OR of 5.52 (95% CI = 2.33–13.09) for URS (Figure 6J).

We also evaluated the independent diagnostic efficacy of the key genes LTB and TCP11L2 across multiple datasets. Their AUC values were greater than 0.8 in several datasets, indicating relatively good diagnostic value (Supplementary Figure S4). The meta-analysis showed a pooled OR of 0.3 (95% CI = 0.08–1.10) for LTB and a pooled OR of 2.33 (95% CI = 1.05–5.18) for TCP11L2 (Supplementary Figure S5).

### Ubiquitination characteristics of sepsis at the single-cell level

**FIGURE 7:**
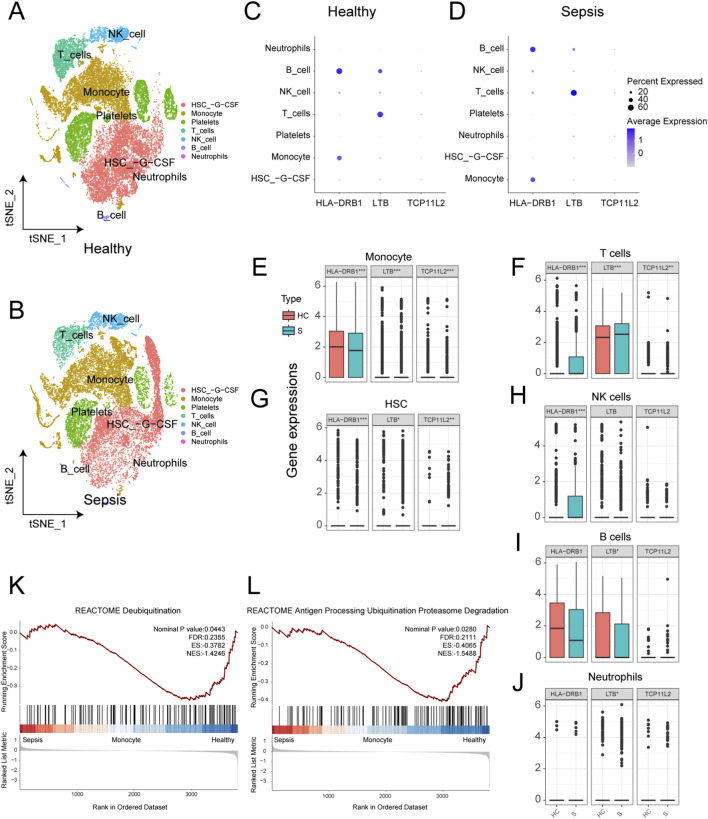
Ubiquitination characteristics of sepsis at the single-cell level. **(A)** t-SNE plot of the distribution of various cell types in normal samples. **(B)** t-SNE plot of the distribution of various cell types in sepsis samples. **(C)** Bubble chart of the expression of three key genes in different cell types in normal samples. **(D)** Bubble chart of the expression of three key genes in different cell types in sepsis samples. **(E)** Box plot of the expression differences of three genes in monocytes between normal and sepsis samples. **(F)** Box plot of the expression differences of three genes in T cells between normal and sepsis samples. **(G)** Box plot of the expression differences of three genes in HSC between normal and sepsis samples. **(H)** Box plot of the expression difference of HLA-DRB1 in NK cells between normal and sepsis samples. **(I)** Box plot of the expression difference of LTB in B cells between normal and sepsis samples. **(J)** Box plot of the expression difference of LTB in neutrophils between normal and sepsis samples. **(K)** GSEA enrichment analysis showing significant enrichment of the REACTOME Deubiquitylation pathway in sepsis samples. **(L)** GSEA enrichment analysis showing significant enrichment of the REACTOME Antigen Processing Ubiquitination and Proteasome Degradation pathway in sepsis samples.

We analyzed a total of 27,808 single-cell samples from five healthy control subjects and 21,644 single-cell samples from four sepsis patients. After automated cell annotation using SingleR, seven cell types were identified: hematopoietic stem cells (HSCs), monocytes, platelets, T cells, natural killer (NK) cells, B cells, and neutrophils (Figures 7A,B). We then examined the expression of three key genes across these cell types in both healthy and sepsis samples. All three genes were expressed to varying degrees in all cell types except platelets (Figures 7C,D). Differential expression analysis at the single-cell level revealed that all three genes were significantly differentially expressed in monocytes, T cells, and HSCs (Figures 7E–G). In NK cells, HLA-DRB1 showed significant differential expression (Figure 7H); in B cells, LTB exhibited significant differences (Figure 7I); and in neutrophils, LTB also showed significant differences (Figure 7J). Furthermore, GSEA enrichment analysis between normal and sepsis samples showed significant enrichment in the REACTOME Deubiquitylation pathway (Figure 7K) and the REACTOME Antigen Processing: Ubiquitination and Proteasome Degradation pathway (Figure 7L), suggesting that dysregulation of ubiquitination processes may be associated with sepsis at the immune cell level.

We also calculated the URS score at the single-cell level. For each cell, we computed the URS based on single-cell transcriptomic data, thereby identifying cells with different URS states. As shown in the tSNE plot of sepsis samples (Supplementary Figure S6A), only a small fraction of cells were classified as high-URS-risk, with neutrophils exhibiting the highest URS scores (Supplementary Figure S6B). Pathway enrichment analysis revealed that REACTOME Antigen Processing: Ubiquitination and Proteasome Degradation and other E3 ubiquitination-related pathways were highly enriched in monocytes and NK cells under high URS conditions (Supplementary Figure S6C,D), suggesting that ubiquitination may play a pivotal role in antigen processing and presentation. Further KEGG enrichment analyses in monocytes (Supplementary Figure S6E) and NK cells (Supplementary Figure S6F) highlighted the top 30 enriched pathways, many of which were closely associated with E3 ubiquitination processes, including Ubiquitin mediated proteolysis, Proteasome, and Protein processing in endoplasmic reticulum (Kanehisa et al., 2016). These findings suggest that the ubiquitination system may play a multilayered regulatory role in inflammation, protein degradation, endoplasmic reticulum homeostasis, and antigen presentation, representing a key mechanism underlying immune dysfunction in sepsis.

### Validation of candidate genes in sepsis patients and healthy controls

**FIGURE 8:**
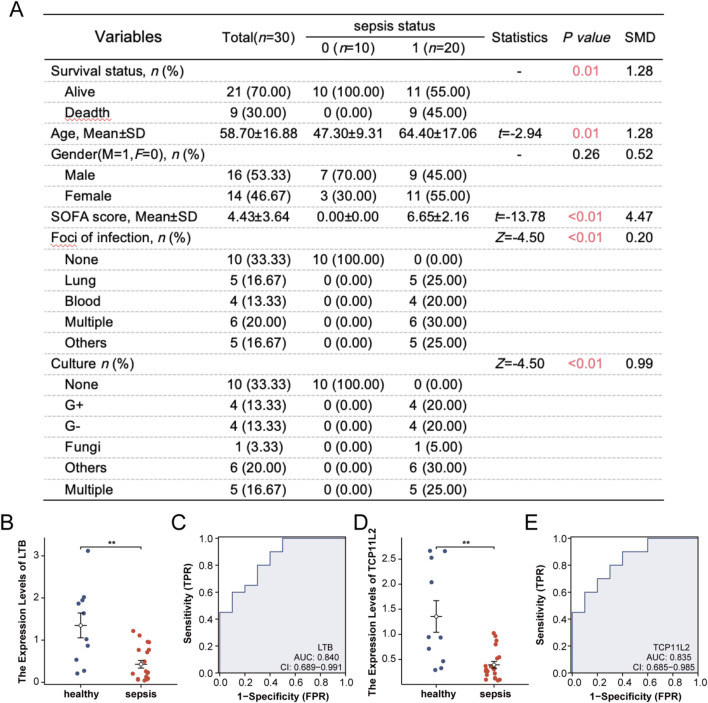
Validation of candidate genes in sepsis patients and healthy controls. **(A)** Baseline clinical characteristics of sepsis patients and healthy controls. **(B)** Relative expression levels of LTB measured by RT-qPCR. **(C)** ROC curve assessing the diagnostic performance of LTB expression for sepsis. **(D)** Relative expression levels of TCP11L2 measured by RT-qPCR. **(E)** ROC curve assessing the diagnostic performance of TCP11L2 expression for sepsis.

We prospectively collected blood samples from 20 sepsis patients and 10 healthy controls. Baseline characteristics of the two groups are shown in Figure 8A. RT-qPCR analysis revealed that the expression level of LTB was significantly downregulated in sepsis patients compared with healthy controls (Figure 8B). Receiver operating characteristic (ROC) analysis demonstrated that LTB expression had good discriminatory power for distinguishing sepsis patients from controls (Figure 8C). Similarly, TCP11L2 expression was markedly reduced in sepsis patients (Figure 8D), and ROC curve analysis further confirmed its diagnostic value (Figure 8E).

## Discussion

This study was designed to test the hypothesis that E3 ubiquitin ligase-related genes could be used to develop a prognostic classifier for sepsis. We comprehensively investigated the role of these genes in sepsis by integrating multiple transcriptomic datasets and applying machine learning algorithms, we established a ubiquitination-related risk score (URS) that robustly stratified patients into prognostic groups. The reliability of the URS was confirmed across public GEO cohorts and an independent clinical cohort. Moreover, we identified two key genes, LTB and TCP11L2, that were consistently downregulated in sepsis and demonstrated significant diagnostic value validated by RT-qPCR. Together, these findings collectively support our initial hypothesis and underscore a significant association of ubiquitination-related pathways with sepsis and provide potential biomarkers for clinical translation.

The decision to focus on the ubiquitination system, and specifically E3 ligase-related genes, was driven by its position as a master regulator at the nexus of multiple sepsis-relevant pathways. Beyond its canonical role in protein degradation, ubiquitination intricately controls immune cell activation, inflammatory signaling transduction, cellular stress responses, and antigen presentation — processes all profoundly disrupted in sepsis. E3 ligases confer substrate specificity, making their expression patterns potential sentinels of specific pathological cellular states. Therefore, we postulated that a prognostic signature derived from this system would not merely be a correlative marker but could also reflect core dysregulated biological programs, offering both predictive utility and mechanistic insights. However, most investigations have focused on mechanistic pathways in experimental models. Our study aims to extend this knowledge by performing a large-scale bioinformatic analysis combined with clinical validation, as an initial step towards bridging the gap between molecular regulation and translational application. Compared with existing prognostic models based on clinical variables or limited molecular signatures, the URS showed improved robustness and consistency across independent datasets. The multi-level validation strategy, including external transcriptome data, single-cell RNA-seq, and RT-qPCR in patient samples, represents a major strength of this study.

Our systematic screening nominated LTB and TCP11L2 as high-priority candidate genes, whose downregulation provides additional biological insight into sepsis pathophysiology. LTB (Sudhamsu et al., 2013), a member of the TNF superfamily, is involved in lymphoid organogenesis and adaptive immune responses, and its downregulation in sepsis may reflect impaired immune surveillance and T/B cell signaling. TCP11L2, although less well characterized, has been linked to ubiquitination-related processes and protein homeostasis, suggesting that its reduced expression could contribute to defective immune cell activation and dysregulated inflammation. These findings highlight a strong association between the expression of these genes and sepsis outcomes, and motivate future functional studies to determine whether they play a causal role in disturbances of E3 ubiquitin ligase–mediated pathways may underlie key aspects of immune dysfunction during sepsis.

From a clinical perspective, the URS and its component genes may serve as practical tools for early diagnosis and risk stratification in sepsis. Compared with established clinical biomarkers for sepsis, such as procalcitonin (PCT) and C-reactive protein (CRP), URS which derived from a mechanism-oriented gene set (E3 ligases), offers a distinct and potentially complementary value proposition. First, it is explicitly designed as a prognostic risk score, demonstrating stable performance in stratifying mortality risk across independent cohorts (Figure 5). Second, the transcriptomic nature of URS provides a snapshot of the host’s dysregulated molecular state, offering insights into underlying immune pathways (e.g., ubiquitin-proteasome system, antigen presentation) that are not directly captured by protein-level inflammatory markers. In terms of clinical feasibility, measurement of LTB and TCP11L2 expression via RT-qPCR is technically straightforward, though currently less rapid than point-of-care PCT tests. The most immediate clinical application may therefore lie not in replacement, but in integration. Integration of URS with clinical variables further enhanced predictive performance, as demonstrated by our Nomogram model. Such tools may aid clinicians in identifying high-risk patients (Martínez-Paz et al., 2021), guiding therapeutic decisions, and potentially monitoring treatment responses. Furthermore, the involvement of ubiquitination pathways raises the possibility that targeted modulation of E3 ligases or their substrates could represent a novel therapeutic avenue in sepsis.

Several limitations of our study should be acknowledged. First and foremost, our findings are based on observational correlations derived from transcriptomic data and clinical validation. While we have identified LTB and TCP11L2 as promising prognostic biomarkers and validated their differential expression, this study lacks direct functional experiments (e.g., gene knockdown or overexpression in immune cells) to establish a causal relationship between these genes and sepsis pathogenesis or to elucidate their precise mechanistic roles within the ubiquitination system. Future studies are required to bridge this gap. Second, although our analysis included multiple GEO datasets and an independent clinical cohort, the sample size for RT-qPCR validation remained modest. Third, sepsis is a heterogeneous syndrome, and such factors are known to influence outcomes and host responses. But this initial investigation focused on identifying a common ubiquitination-related prognostic signature across unselected sepsis patients, which may reflect a core dysregulated pathway shared across diverse etiologies. Our study did not perform stratified analyses based on specific sepsis etiologies (e.g., bacterial vs. fungal) or infection sites. While we observed an association between URS and pneumonia status in some data, the limited granularity of clinical metadata (particularly microbiological data) in the public datasets prevented a systematic evaluation of the model’s performance across defined subtypes. Future validation in prospectively collected cohorts with detailed clinical and microbiological phenotyping is needed to determine the generalizability and potential subtype-specific utility of the URS. Fourth, the complexity of sepsis pathophysiology suggests that additional molecular layers, such as epigenetic modifications or proteomic alterations, should be integrated into future models.

The promising yet preliminary nature of our findings outlines clear priorities for future research. Future studies should aim to validate the URS and its key genes in larger, prospective, multicenter cohorts and to explore their utility in predicting treatment response or guiding immunomodulatory therapy. Mechanistic experiments focusing on how LTB and TCP11L2 influence immune cell function and ubiquitination networks will be essential. In conclusion, this study highlights ubiquitination-related genes as critical determinants of sepsis outcome, establishes a robust prognostic model, and proposes potential diagnostic biomarkers, thereby providing a foundation for future translational and therapeutic research in sepsis.

## Data Availability

The datasets presented in this study can be found in online repositories. The names of the repository/repositories and accession number(s) can be found in the article/Supplementary Material.

## Supplementary material

SUPPLEMENTARY FIGURE S1 Clinical information heatmaps of different URS groups. **(A)** Clinical information heatmaps of different URS groups in GSE65682. **(B)** Clinical information heatmaps of different URS groups in GSE95233. **(C)** Clinical information heatmaps of different URS groups in GSE294985.

SUPPLEMENTARY FIGURE S2 Univariable and multivariable Cox regression analyses of different datasets. **(A)** The forest plot of univariable Cox regression analysis in GSE65682. **(B)** The forest plot of multivariable Cox regression analysis in GSE65682. **(C)** The forest plot of univariable Cox regression analysis in GSE95233. **(D)** The forest plot of multivariable Cox regression analysis in GSE95233. **(E)** The forest plot of univariable Cox regression analysis in GSE294985. **(F)** The forest plot of multivariable Cox regression analysis in GSE294985.

SUPPLEMENTARY FIGURE S3 Construction of a clinical Nomogram model. **(A)** Nomogram modeling in the GSE65682 dataset. **(B)** Calibration curve of the Nomogram model. **(C)** ROC curve for the prognostic prediction ability of the URS score. **(D)** ROC curve for the prognostic prediction ability of the Nomogram score.

SUPPLEMENTARY FIGURE S4 Assessment of the Diagnostic Efficacy of LTB for Sepsis. **(A)** The diagnostic ROC curve of LTB for sepsis in GSE65682. **(B)** The diagnostic ROC curve of LTB for sepsis in GSE95233. **(C)** The diagnostic ROC curve of LTB for sepsis in GSE131761. **(D)** The diagnostic ROC curve of LTB for sepsis in GSE137340. **(E)**. The diagnostic ROC curve of LTB for sepsis in GSE236713. **(F)** The diagnostic ROC curve of LTB for sepsis in GSE28750. **(G)** The diagnostic ROC curve of LTB for sepsis in GSE57065. **(H)** The diagnostic ROC curve of LTB for sepsis in GSE69528. **(I)** The diagnostic ROC curve of LTB for sepsis in GSE294985. **(J)** Meta-analysis of the diagnostic efficacy of LTB for sepsis.

SUPPLEMENTARY FIGURE S5 Assessment of the Diagnostic Efficacy of TCP11L2 for Sepsis. **(A)** The diagnostic ROC curve of TCP11L2 for sepsis in GSE65682. **(B)** The diagnostic ROC curve of TCP11L2 for sepsis in GSE95233. **(C)** The diagnostic ROC curve of TCP11L2 for sepsis in GSE131761. **(D)** The diagnostic ROC curve of TCP11L2 for sepsis in GSE137340. **(E)** The diagnostic ROC curve of TCP11L2 for sepsis in GSE236713. **(F)** The diagnostic ROC curve of TCP11L2 for sepsis in GSE28750. **(G)** The diagnostic ROC curve of TCP11L2 for sepsis in GSE57065. **(H)** The diagnostic ROC curve of TCP11L2 for sepsis in GSE69528. **(I)** The diagnostic ROC curve of TCP11L2 for sepsis in GSE294985. **(J)** Meta-analysis of the diagnostic efficacy of TCP11L2 for sepsis.

SUPPLEMENTARY FIGURE S6 URS states and associated pathway enrichment at the single-cell level. **(A)** t-SNE plot of cells from sepsis samples, with cells colored by URS score. **(B)** URS scores across different immune cell types, with neutrophils exhibiting the highest scores. **(C)** GSEA enrichment analysis of monocytes under different URS states. **(D)** GSEA enrichment analysis of NK cells under different URS states. **(E)** KEGG enrichment analysis of monocytes in the high-URS group, displaying the top 30 enriched pathways. **(F)** KEGG enrichment analysis of NK cells in the high-URS group, displaying the top 30 enriched pathways.
