# Supplementary figures and images for: Construction of a robust sepsis prognostic classifier based on E3 ubiquitin ligase-related genes
Source: Front Mol Biosci. 2026 Feb 17;13:1726356. doi: 10.3389/fmolb.2026.1726356 (PMC12953455; doi:10.3389/fmolb.2026.1726356)

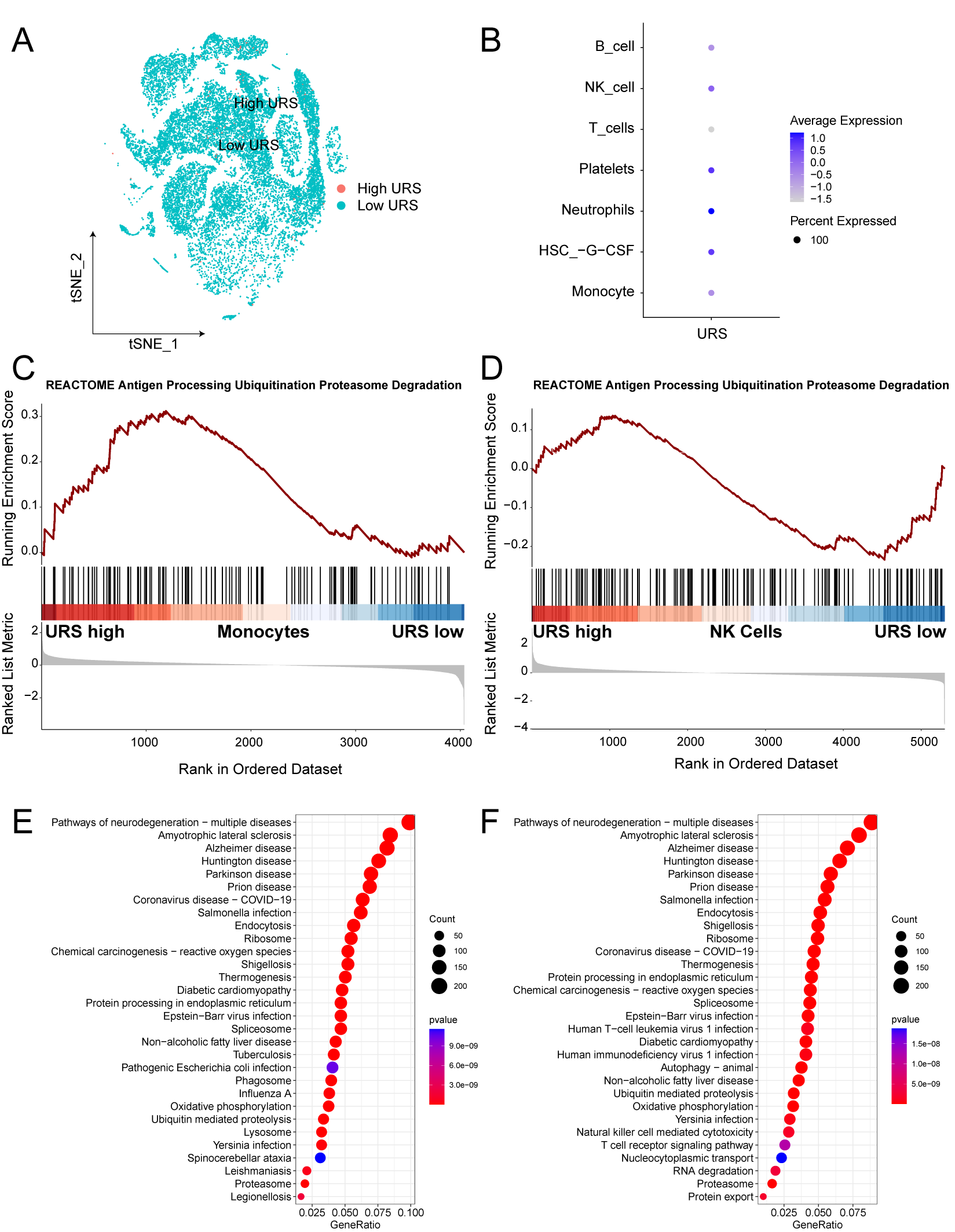

Supplement: Supplementary file 3 [file Image6.tif]

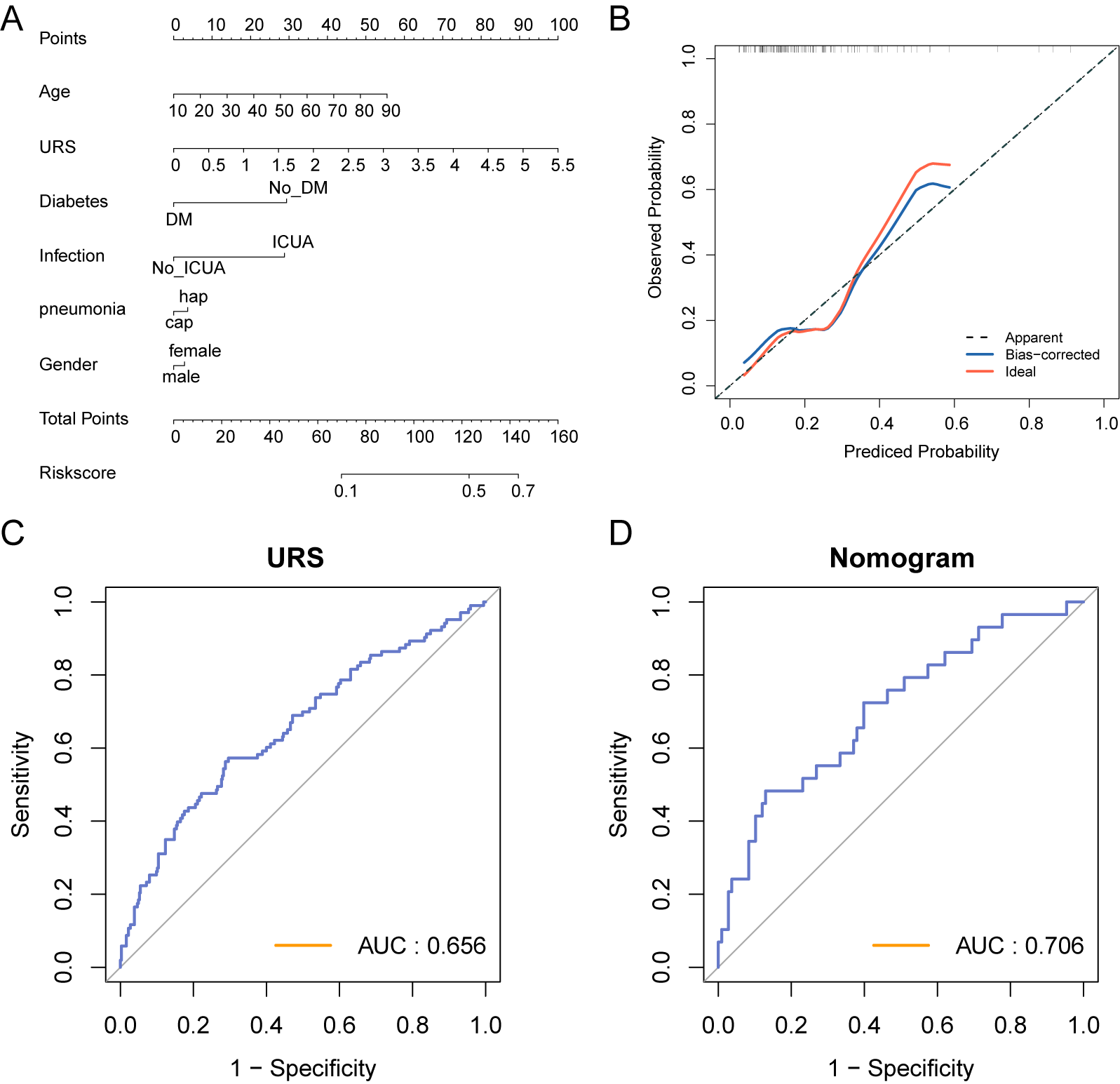

Supplement: Supplementary file 4 [file Image3.tif]

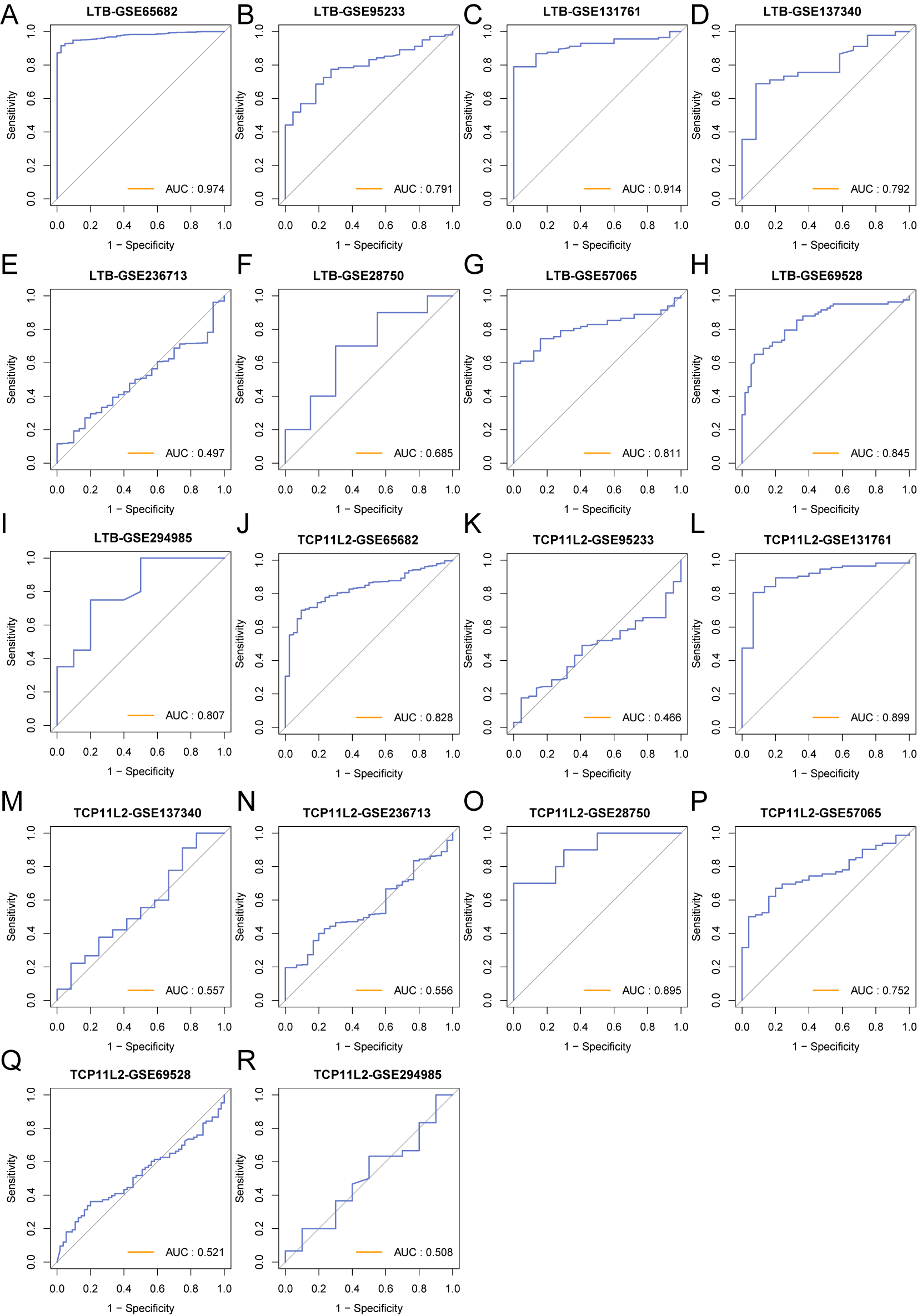

Supplement: Supplementary file 5 [file Image4.tif]

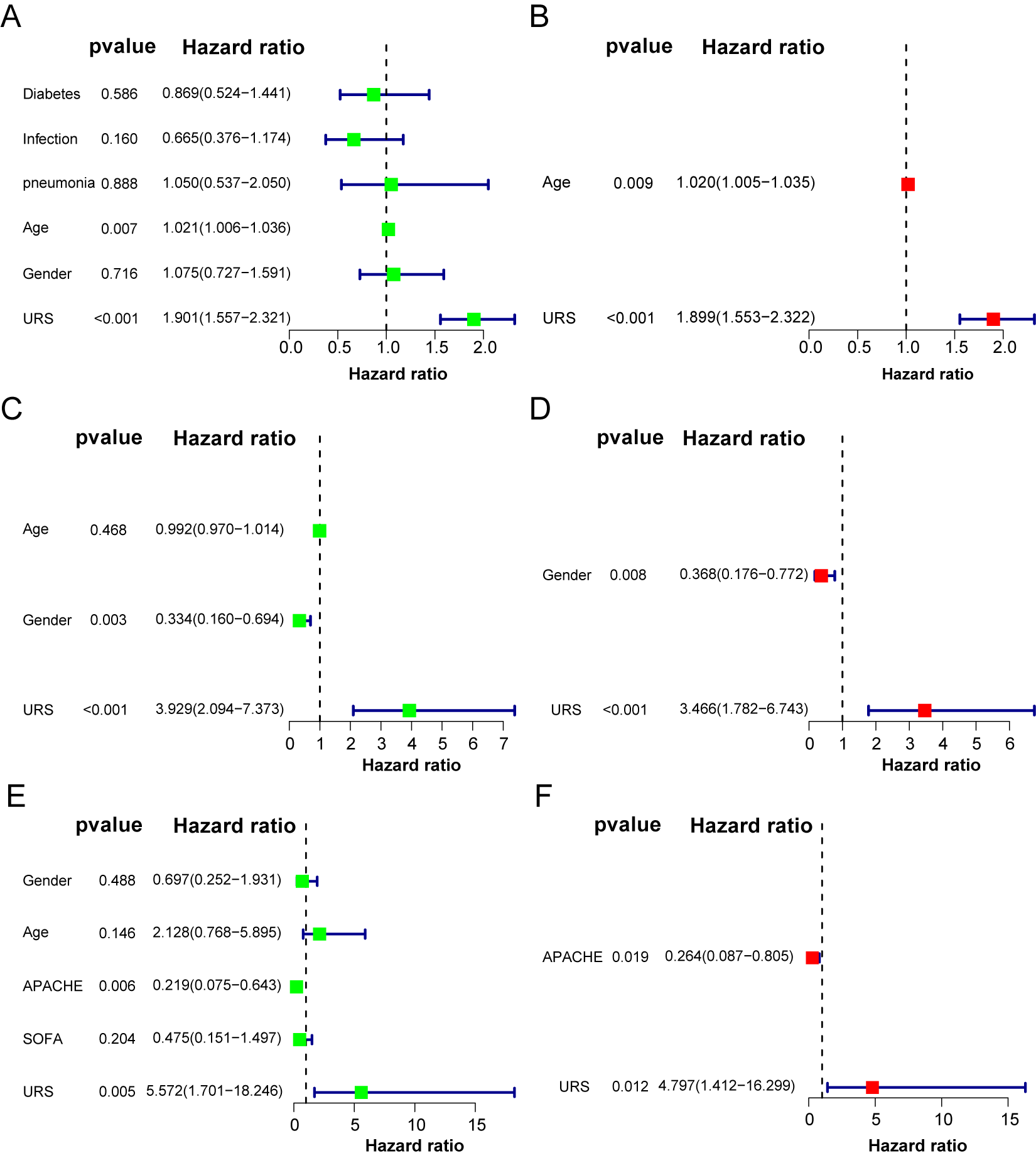

Supplement: Supplementary file 6 [file Image2.tif]

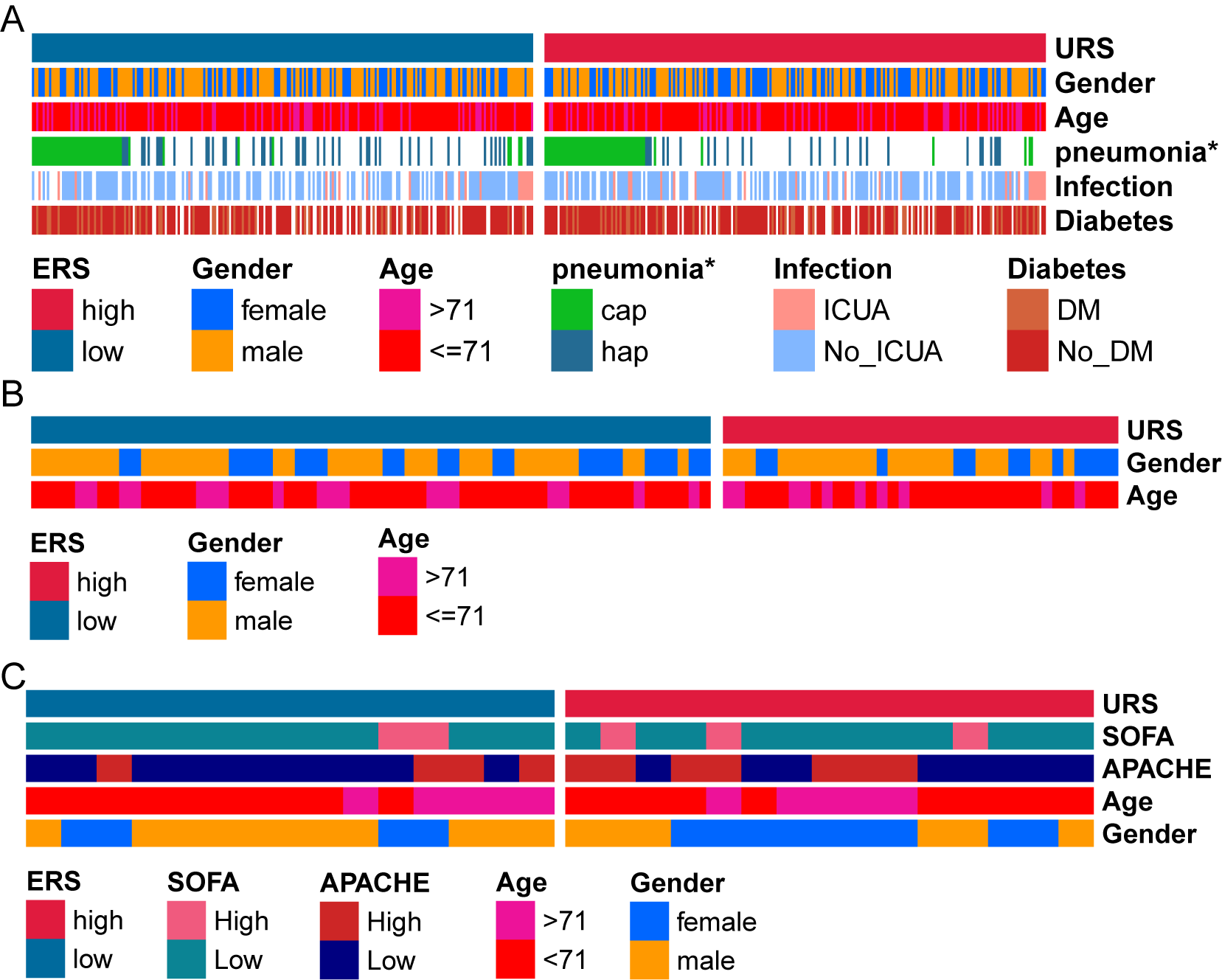

Supplement: Supplementary file 7 [file Image1.tif]

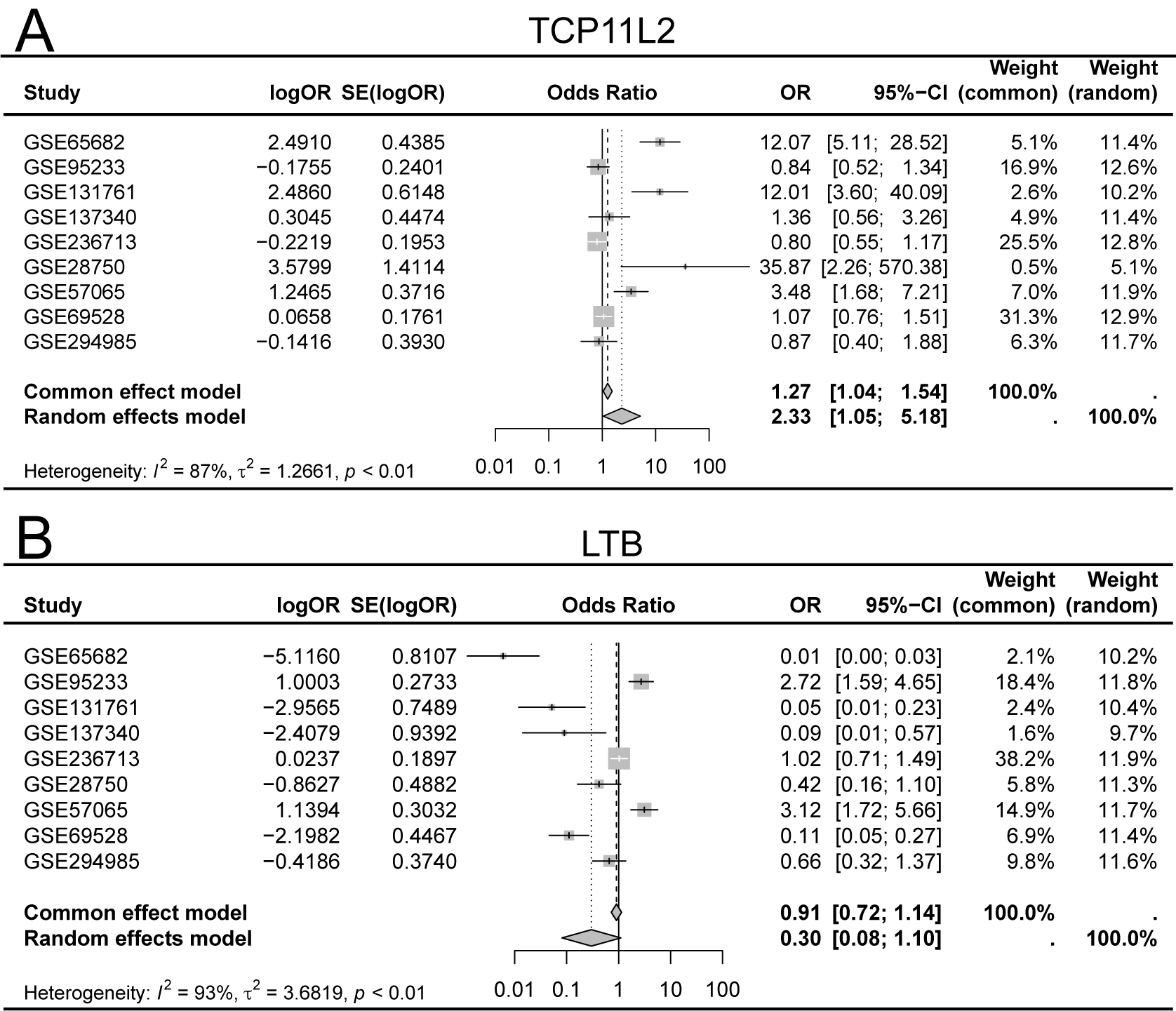

Supplement: Supplementary file 9 [file Image5.tif]
